# Validating the accuracy of real-time phase-contrast MRI and quantifying the effects of free breathing on cerebrospinal fluid dynamics

**DOI:** 10.1186/s12987-024-00520-0

**Published:** 2024-03-07

**Authors:** Pan Liu, Kimi Owashi, Heimiri Monnier, Serge Metanbou, Cyrille Capel, Olivier Balédent

**Affiliations:** 1 CHIMERE UR 7516, Jules Verne University of Picardy, Amiens, 80000 France; 2 Medical Image Processing Department, Amiens Picardy University Medical Center, Amiens, 80000 France; 3 Radiology Department, Amiens Picardy University Medical Center, Amiens, 80000 France; 4 Neurosurgery Department, Amiens Picardy University Medical Center, Amiens, 8000 France

**Keywords:** Real-time phase contrast MRI, Phase contrast MRI, Breathing effect, Cerebrospinal fluid, Cerebral circulation

## Abstract

**Background:**

Understanding of the cerebrospinal fluid (CSF) circulation is essential for physiological studies and clinical diagnosis. Real-time phase contrast sequences (RT-PC) can quantify beat-to-beat CSF flow signals. However, the detailed effects of free-breathing on CSF parameters are not fully understood. This study aims to validate RT-PC’s accuracy by comparing it with the conventional phase-contrast sequence (CINE-PC) and quantify the effect of free-breathing on CSF parameters at the intracranial and extracranial levels using a time-domain multiparametric analysis method.

**Methods:**

Thirty-six healthy participants underwent MRI in a 3T scanner for CSF oscillations quantification at the cervical spine (C2-C3) and Sylvian aqueduct, using CINE-PC and RT-PC. CINE-PC uses 32 velocity maps to represent dynamic CSF flow over an average cardiac cycle, while RT-PC continuously quantifies CSF flow over 45-seconds. Free-breathing signals were recorded from 25 participants. RT-PC signal was segmented into independent cardiac cycle flow curves (Q_t_) and reconstructed into an averaged Q_t_. To assess RT-PC’s accuracy, parameters such as segmented area, flow amplitude, and stroke volume (SV) of the reconstructed Q_t_ from RT-PC were compared with those derived from the averaged Q_t_ generated by CINE-PC. The breathing signal was used to categorize the Q_t_ into expiratory or inspiratory phases, enabling the reconstruction of two Q_t_ for inspiration and expiration. The breathing effects on various CSF parameters can be quantified by comparing these two reconstructed Qt.

**Results:**

RT-PC overestimated CSF area (82.7% at aqueduct, 11.5% at C2-C3) compared to CINE-PC. Stroke volumes for CINE-PC were 615 mm³ (aqueduct) and 43 mm³ (spinal), and 581 mm³ (aqueduct) and 46 mm³ (spinal) for RT-PC. During thoracic pressure increase, spinal CSF net flow, flow amplitude, SV, and cardiac period increased by 6.3%, 6.8%, 14%, and 6%, respectively. Breathing effects on net flow showed a significant phase difference compared to the other parameters. Aqueduct-CSF flows were more affected by breathing than spinal-CSF.

**Conclusions:**

RT-PC accurately quantifies CSF oscillations in real-time and eliminates the need for cardiac synchronization, enabling the quantification of the cardiac and breathing components of CSF flow. This study quantifies the impact of free-breathing on CSF parameters, offering valuable physiological references for understanding the effects of breathing on CSF dynamics.

**Supplementary Information:**

The online version contains supplementary material available at 10.1186/s12987-024-00520-0.

## Background

Cerebrospinal fluid (CSF) is essential for the homeostasis of the central nervous system, acting as a cushion for the brain and spinal cord and assisting in the removal of waste products [1–5]. Disturbances in CSF micro-circulation can have profound effects on brain function and are associated with various neurological and neurodegenerative disorders [6, 7]. It is also interesting to point out that protein concentration in the brain is not only affected by micro-circulation from the glymphatic system but also by other factors. The total protein concentration is 2.5 times higher in the lumbar CSF than the ventricular CSF due to the gradual influx of proteins moving from the choroid plexus to the lumbar spinal canal [8]. However, the concentration of proteins synthesized in the brain can sometimes even be lower in the lumbar region than in the ventricular region, as observed with tau protein [9]. Protein concentrations are not uniform in the different CSF compartments also due to the amplitude of the macro-CSF flow oscillations, which can vary with age [10] and pathologies [11, 12] and play a shaker role in the CSF [13].

Therefore, precise quantification and understanding of macro and micro CSF dynamics are essential not only for diagnosing and management of conditions such as hydrocephalus, Alzheimer’s disease and Chiari malformations [14–17] but also for the overall understanding of brain physiology.

Conventional cine phase contrast MRI (CINE-PC) [18, 19], currently considered the gold standard for quantifying CSF flow, relies on cardiac gating and requires several cardiac cycles to reconstruct an average cardiac cycle flow curve (averaged Q_t_). As a result, it primarily quantifies the contributions of cardiac pulsations to CSF hydrodynamics [20–23].

However, in addition to cardiac pulsations, breathing can also affect CSF oscillations [24–28]. The temporal acquisition limitations of CINE-PC make it unsuitable for studying the effect of breathing on CSF hydrodynamics. Real-time phase-contrast MRI (RT-PC) can quantify the continuous Q_t_ of blood or CSF with a temporal resolution of hundreds of ms without cardiac synchronizers. Although available as early as the 1990s [29, 30], the performance of the equipment at that time resulted in low spatial resolution for cerebral circulation quantification.

Recently, an increasing number of studies have begun using RT-PC to investigate how breathing affects CSF circulation [31–37]. These studies have examined different breathing patterns and revealed changes in CSF flow rate, including increased caudocranial flow during inspiration and increased craniocaudal flow during expiration. Most of these studies use frequency domain analysis methods that quantify the effect of breathing on CSF by comparing the ratio of breathing spectrum components to cardiac spectrum components. This method primarily observes the effects of breathing on CSF flow rate. However, in addition to flow rate, other critical parameters within the CSF, such as the flow rate amplitude, cardiac period, and average stroke volume, have not been fully explored. For example, the average stroke volume, a key clinical diagnostic parameter representing the volume of CSF oscillations at a given cross-sectional area in a single cardiac cycle, impacted by various neurological disorders [11, 38–41].

Given the significantly lower velocity of CSF flow compared to the flows in the internal carotid artery and jugular vein, this results in a lower signal-to-noise ratio (SNR) [42]. Although using a lower spatial resolution can enhance the SNR and increase the temporal resolution, it might lead to the overestimation of the segmented area due to partial volume effect artifacts [43], particularly when quantifying the CSF at the aqueduct within the intracranial space. Therefore, it is important to validate the accuracy of RT-PC in quantifying CSF before its clinical application. While several studies have focused on the effects of controlled or specific breathing patterns on CSF dynamics, the effects of free breathing on various CSF parameters remain less clear.

This study has two aims. Firstly, it aims to assess the accuracy of RT-PC in measuring CSF flow dynamics by comparing its results with those of the established gold standard, CINE-PC. Secondly, and more importantly, the study aims to quantify the effect of free breathing (hereafter ‘breathing effects’) on both intracranial and extracranial CSF dynamics. This will be achieved by detailed time-domain multi-parametric analysis focusing on multiple flow parameters.

## Methods

### Participants

This study was approved by the local investigational review board (CPP Nord Ouest II, Amiens, France; reference: PI2019_843_0056) and was performed in accordance with the Declaration of Helsinki.

The study population comprised 36 healthy adult participants, 17 females and 19 males, with a mean age of 26 ± 3.8 years and an age range of 19–35 years. The MRI examination lasted about 30 min. All participants were informed of the objectives and procedures of the study. All subjects signed a written informed consent. Exclusion criteria were contraindications for MRI and history of cerebrovascular or respiratory disease.

### Image acquisition

A 3T MRI system (Philips Achieva; maximum gradient = 80 mT/m; slew rate = 120 mT m^− 1^ ms^− 1^) equipped with a 32-channel head coil was used to acquire images of participants in the supine position.

We measured CSF flow at the C2-C3 cervical spinal level and within the aqueduct of Silvius twice, first using CINE-PC and then with RT-PC at the same localization.

**Fig. 1:**
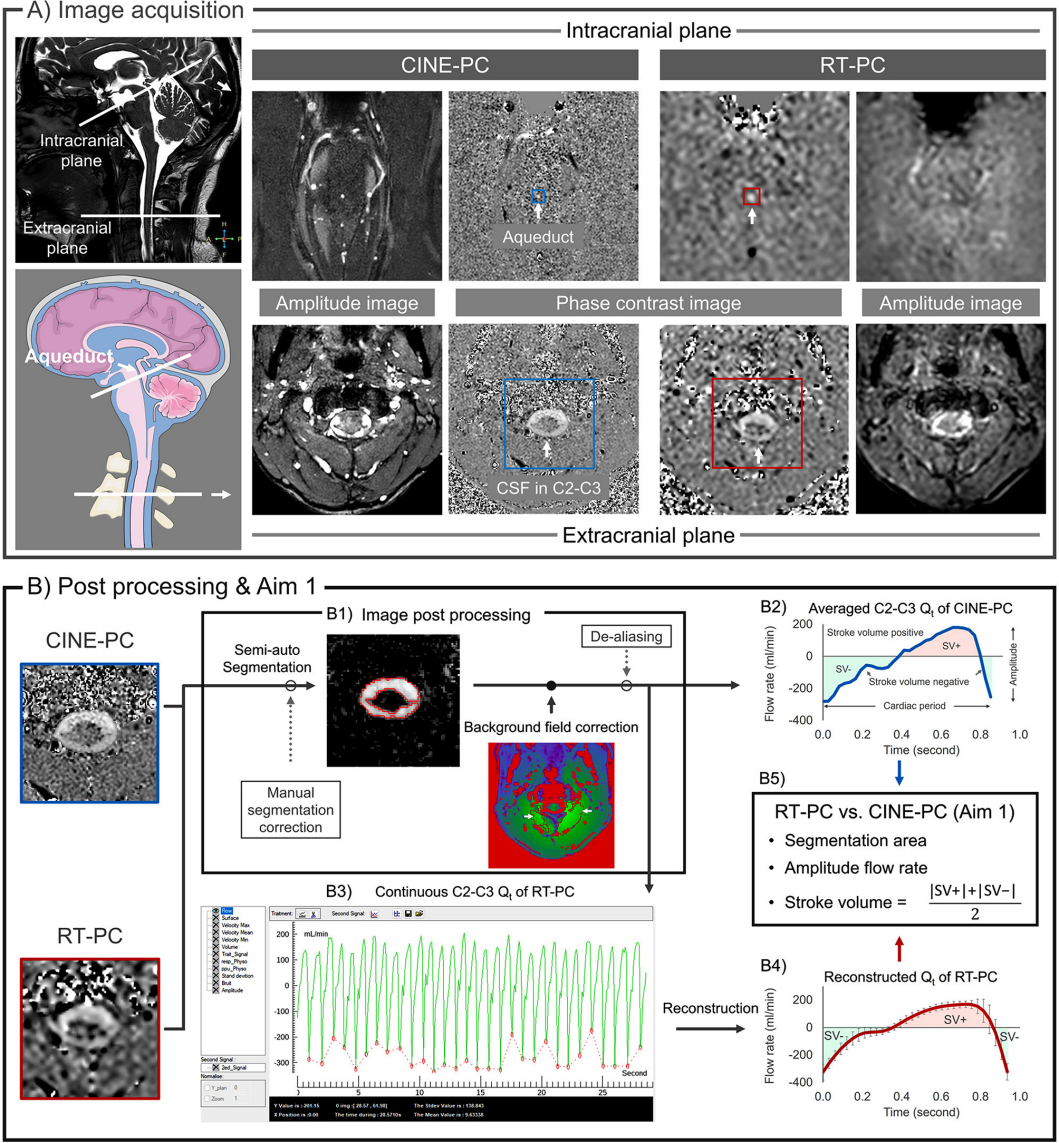
Image acquisition (**A**) and image processing (**B**) for CINE-PC and RT-PC. (**A**) At the intracranial plane, CSF at the aqueduct is measured, while CSF at C2-C3 is measured at the extracranial plane. Amplitude images and phase-contrast images for both CINE-PC (left) and RT-PC (right) are presented. The FOV of RT-PC images was aligned with that of CINE-PC images for ease of comparison. (**B**) CSF post-processing and the first aim procedure at C2-C3 as an example. After the post-processing procedure (**B1**), flow rate signals (Q_t_) were obtained from CINE-PC and RT-PC (**B2** and **B3**). The minimum values in each cardiac cycle (red points in **B3**) were used to segment the continuous Q_t_ into multiple Q_t_. Then, all Q_t_ were used to obtain the reconstructed Q_t_ (**B4**). Finally, the differences in each parameter between the averaged Q_t_ of CINE-PC and the reconstructed Q_t_ of RT-PC were compared (**B5**). SV denotes average stroke volume

The acquisition planes were localized using a sagittal 3D balanced gradient echo sequence (Fig. 1A, top left). The parameters were set as follows: TR = 5.5 ms, TE = 2.2 ms, FOV = 180 × 180 mm^2^, spatial resolution of acquisition = 0.6 × 0.6 × 1.2 mm^3^, and flip angle = 45°.

Subsequently, both CINE-PC and RT-PC used a Cartesian trajectory with a parallel acquisition technique (sensitivity encoding). The phase contrast images of these two sequences were calculated by subtracting two velocity maps obtained with opposite bipolar gradients (i.e., an opposite-polarity flow-encoded pair). Based on individual differences, the velocity encoding (VENC) was set to either 10 or 20 cm/s for the intracranial plane (aqueduct) and to either 5 or 10 cm/s for the extracranial plane (C2-C3). The direction of flow towards the cranium was defined as positive.

For CINE-PC, the parameters were set as follows: FOV = 90 × 90 mm² (aqueduct) and 120 × 120 mm² (C2-C3); acquisition spatial resolution = 0.5 × 0.5 mm^2^ (aqueduct) and 0.8 × 0.8 mm^2^ (C2-C3); thickness = 2 mm (aqueduct) and 3 mm (C2-C3); sampling time = 56–153 s; flip angle = 30°; TR = 14.7 ~ 14.8 ms; TE = 7.7 ~ 9.3 ms; sensitivity encoding factor = 1.5. A finger plethysmograph was used for cardiac gating. Each CINE-PC acquisition provided 32 phase contrast images.

RT-PC parameters were set as follows: FOV = 140 × 140 mm^2^ for both planes; acquisition spatial resolution = 2 × 2 mm^2^; thickness = 4 mm; flip angle = 10°; TR = 9.8 ~ 12 ms; TE = 5.5 ~ 7.6 ms; imaging speed = 78 ms/image, 87 ms/image, or 96 ms/image depending on VENC; EPI-factor = 7 (7 echoes collected during each TR); sensitivity encoding factor = 2.5. For the first 16 participants, 300 images were acquired, and for the last 20 participants, 500 images were acquired. The breathing signals of 25 participants were measured during acquisition (using a chest belt), and all participants were free-breathing throughout the process.

### Image processing

CINE-PC and RT-PC data were post-processed using in-house Flow software [20, 44, 45]. The Q_t_ extraction process is described as follows (Fig. 1B1):

A semi-automatic segmentation algorithm based on the frequency domain features of pixel velocity was used for CSF delineation [20]. Subsequently, a fully automated process was implemented to correct the background field and remove eddy current artifacts (Fig. S1). For this purpose, stationary tissue regions of interest surrounding the CSF were identified and their average velocity was considered the new reference for zero velocity (Sect. 5.2 in [45]). Furthermore, our software included a de-aliasing correction for instances in which the CSF velocity exceeded the VENCs.

CINE-PC generated an averaged Q_t_ (cardiac cycle flow rate curve) with 32 sampling points for both C2-C3 and the aqueduct, as shown in Fig. 1B2. In contrast, RT-PC provided a continuous Q_t_ with either 300 or 500 sample points over several breathing cycles, depicted in Fig. 1B3.

### Aim 1: RT-PC vs. CINE-PC

To compare RT-PC and CINE-PC, the continuous Q_t_ from RT-PC was reconstructed into a single mean Q_t_ (Fig. 1B3 & Fig. 1B4). First, the software identified the minimum values of each cardiac cycle in the continuous Q_t_ and used them as segmentation points to divide the continuous Q_t_ into multiple independent Q_t_ (Sect. 5.5 in [45]). Next, each Q_t_ was interpolated to increase the number of sampled points to 32. The final step was to average these individual Q_t_ into a reconstructed Q_t_ with 32 sampling points, as in the CINE-PC format.

We computed the following CSF flow parameters to compare the reconstructed Q_t_ of RT-PC with the averaged Q_t_ of CINE-PC (Fig. 1B2 & B5):

Segment area: Segmentation area of the aqueduct and C2-C3 regions.

Q_net_: Net flow rate of the Q_t_. Positive values indicate caudocranial direction.

Amplitude: Amplitude of Q_t_. The difference between the maximum and minimum flow rate values.

T_c_: Cardiac period. The average duration of Q_t_.

SV: The average of positive and negative stroke volume. It represents the volume of fluid oscillating within a cardiac cycle through the acquisition level.

V_Max_ and V_Min_: The caudocranial and craniocaudal peak flow velocities, indicating the average velocities at maximum and minimum Q_t_, respectively.

### Aim 2: quantification of the breathing effects on CSF

**Fig. 2:**
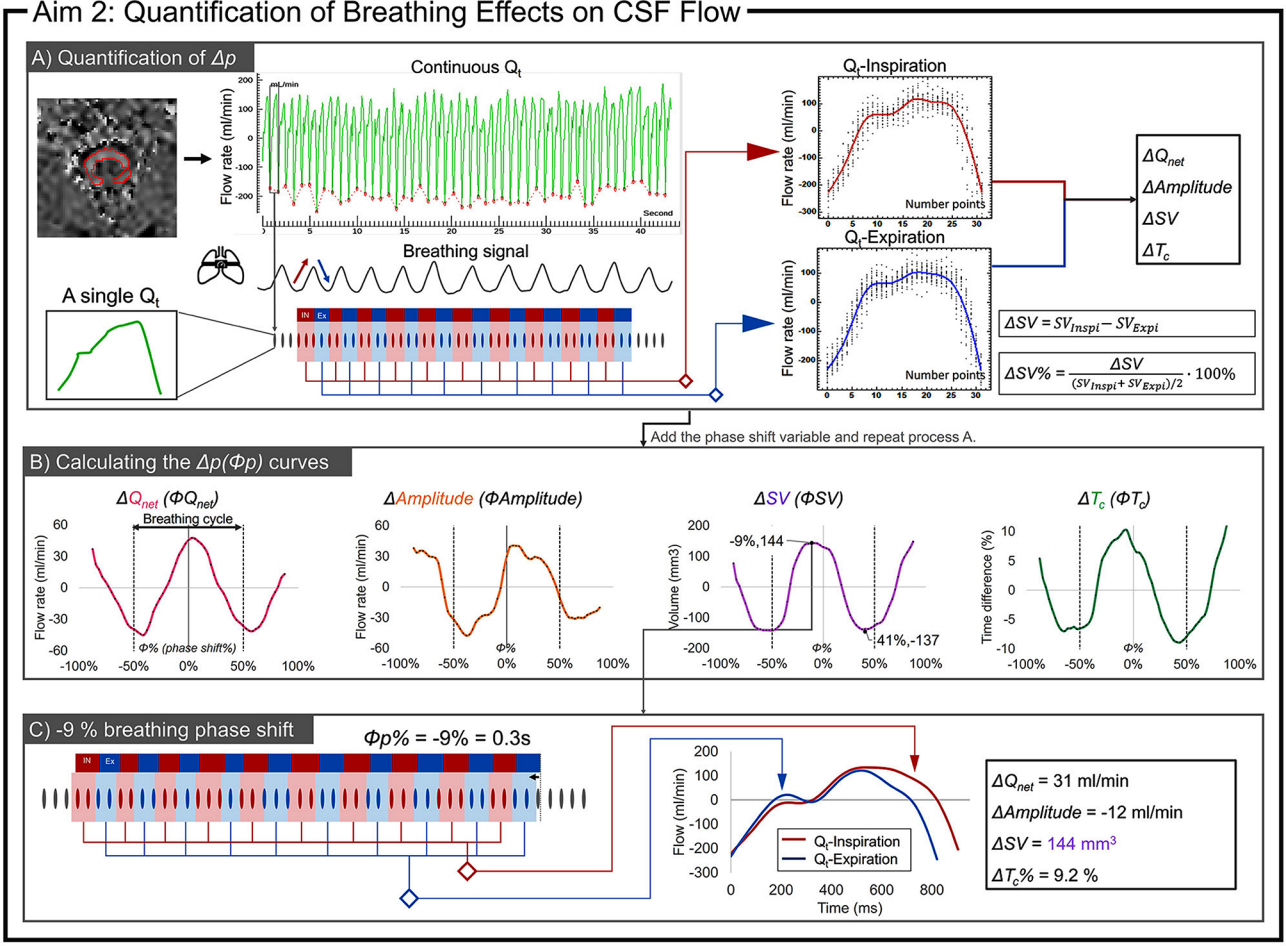
Flow chart for quantifying the breathing effects (*Δp* and *Φp*) on CSF. Definition of the inspiratory phase (IN, red) and the expiratory phase (EX, blue), using the breathing signal. (**A**) Reconstruction of Q_t_-Inspiration (red curve) and Q_t_Expiration (blue curve) from the respective inspiratory Q_t_ (red) and expiratory Q_t_ (blue) points extracted from the continuous Q_t_. Then, *Δp* is calculated for each evaluated parameter with *Φp* = 0. (**B**) The breathing window is shifted from − 3s to + 3s in steps of 0.1 s. The previous steps are repeated to obtain the *Δp(Φp)* curves. (**C**) Represents the *Δp* values for four parameters at *Φp* = -9% (0.3 s). At this point, the *ΔSV* reaches the maximum value

This study used a time-domain multiparameter analysis method to quantitatively investigate breathing effects on CSF. The method was described in a previous study involving 10 participants [46]. The detailed procedures are outlined as follows:

Continuous Q_t_ was segmented into multiple independent Q_t_. The breathing signal was used to delineate the inspiratory and expiratory phases. The inspiratory phase corresponds to the ascending breathing signal, characterized by an increase in thoracic volume (Fig. 2). The midpoint of each independent Q_t_ was used to determine the corresponding breathing phase.

After selecting several complete breathing cycles, all Q_t_ from the inspiratory phases were reconstructed into an average flow curve for the inspiratory phase, labeled as Q_t_-Inspiration. Similarly, the reconstructed Q_t_-Expiration was obtained for the expiratory phase (Fig. 2A).

By comparing the parameters between Q_t_-Inspiration and Q_t_-Expiration, a differential value, *Δp*, was determined, where *p* denotes four parameters: Q_net_, Amplitude, SV, and T_c_. A phase shift variable (*Φp*) was introduced to determine the maximum *Δp*. Considering that the free breathing cycle was usually less than 6 s, the range of *Φp* was set from − 3 s to + 3 s with 0.1 s increments to iterate through the process in Fig. 2A. The *Δp(Φp)* curve was obtained after 60 iterations, representing the *Δp* under varying *Φp*. When CSF flow is influenced by breathing, the frequency of the *Δp(Φp)* curve corresponds to the breathing frequency (Fig. 2B).

We defined the average peak of *Δp(Φp)* curve as the intensity of the breathing effect, labelled *Δp*. We also recorded the corresponding *Φp%* (the percentage of *Φp* relative to the average breathing cycle), which indicates the phase shift where the breathing effect is maximum.

For example, as shown in Fig. 2B and C, when examining the breathing effect on SV, it was observed that the extremes of the *ΔSV(ΦSV)* curve were 144 mm^3^ and − 137 mm^3^ for a *ΦSV* of -9% (-9% indicates that the breathing phases were shifted to the left by 9% of the average breathing cycle, i.e., 3.4 s × 0.09 = 0.3 s) and 41% (1.4 s), respectively. Therefore, the average *ΔSV* = 140.5 mm^3^ was chosen to represent the intensity of breathing effect on SV with a corresponding *ΦSV%* of -9% (closest to 0%).

To facilitate inter-individual comparisons, *Δp* was normalized to percentages, labelled as *Δp%*. This normalization was achieved by dividing *Δp* by the mean value of the corresponding parameter. For Q_net_, which often approaches zero, *ΔQ*_*net*_*%* was calculated by dividing *ΔQ*_*net*_ by the Amplitude. Additionally, *Φp%* was converted into degrees (*Φp°*); for instance, -9% translates to -0.09 × 360° = -32.4°.

### Statistical analysis

Statistical evaluations were conducted using R software. Data are presented as mean ± SD. Data normality was assessed using the Shapiro-Wilk test. Depending on the data distribution, differences between group pairs were analyzed using either Student’s t-test or Wilcoxon’s test. Pearson’s or Spearman’s test was used to assess correlations between groups. The agreement between RT-PC and CINE-PC was quantified using a Bland-Altman plot. All tests were two-tailed with a significance level of *p* < 0.05.

## Results

### Aim 1: RT-PC vs. CINE-PC

**Fig. 3:**
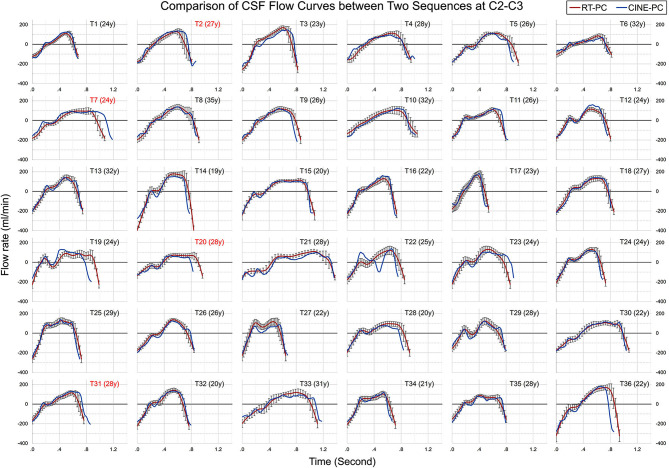
CSF average cardiac cycle flow curves (averaged Q_t_) of CINE-PC (in blue) and reconstructed Q_t_ of RT-PC (in red) at C2-C3 for 36 participants. Each plot is labelled with the participant’s serial number and age. Red-labeled plots indicate a cardiac period difference of more than 10% between the two sequences. For comparison, all plots have consistent axis ranges: y from − 400 to 250 ml/min and x from 0 to 1.4 s

**Fig. 4:**
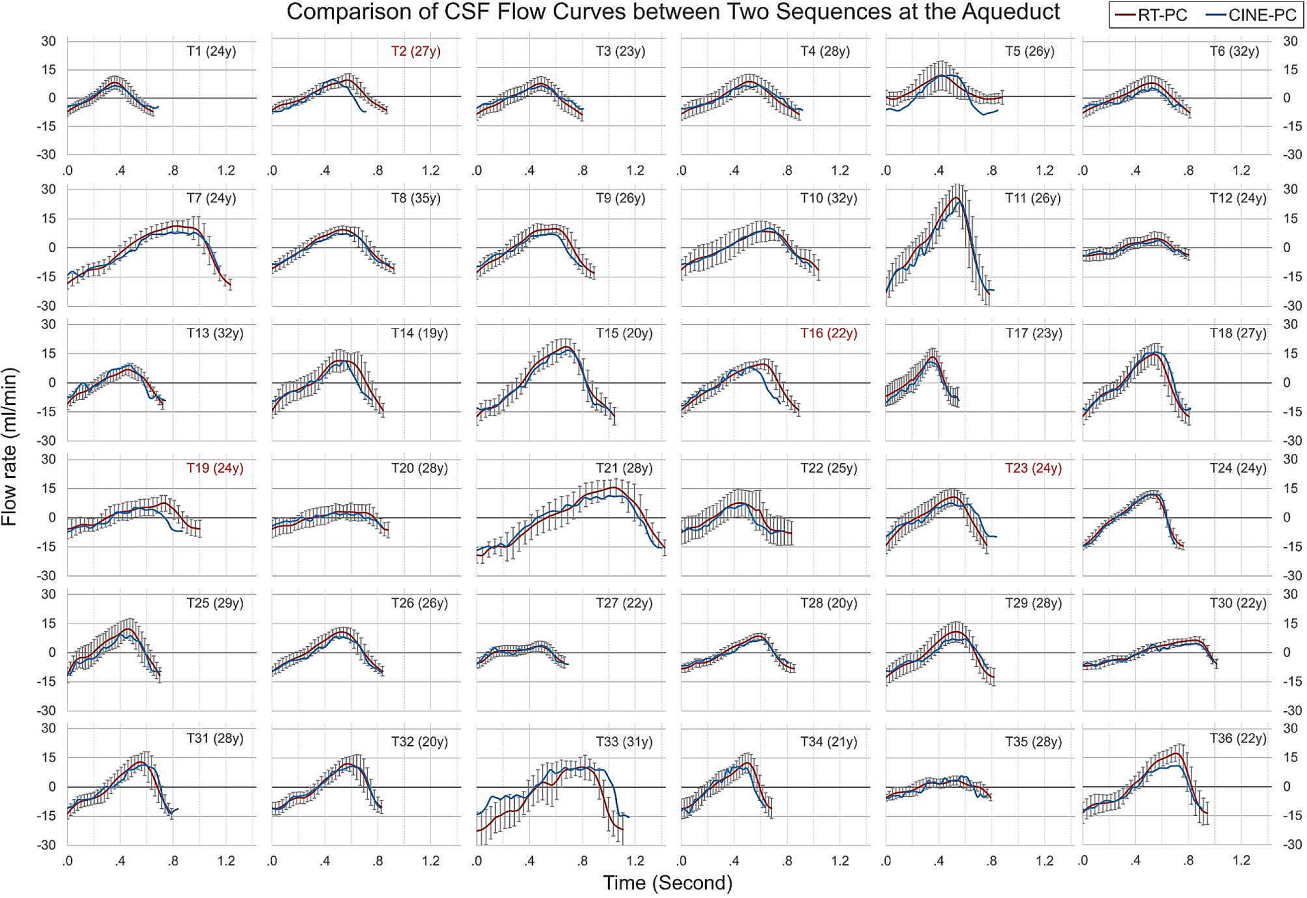
CSF average cardiac cycle flow curves (averaged Qt) of CINE-PC (in blue) and reconstructed Qt of RT-PC (in red) at the aqueduct for 36 participants. Each plot is labeled with the participant’s serial number and age. Red-labeled plots indicate a cardiac period difference of more than 10% between the two sequences. For comparison, all plots have consistent axis ranges: y from − 30 to 33 ml/min and x from 0 to 1.4 s

Figures 3 and 4 show the CSF flow curves of all 36 participants at the C2-C3 and aqueduct planes obtained by CINE-PC and RT-PC. Due to the significant differences in cardiac period (T_c_), four participants whose BPMs differed by > 10% between RT-PC and CINE-PC acquisitions were excluded (marked in red). Data from the final 32 participants were used for Aim 1.

Table 1 presents the values of various parameters derived from the averaged Q_t_ of CINE-PC and the reconstructed Q_t_ of RT-PC. These parameters include Segment Area, Net flow (Q_net_), Amplitude, Stroke volume (SV), Cardiac period (Tc), and peak velocities (V_max_ and V_Min_).

Except for the segment area at the aqueduct and the Q_net_ at both levels, the parameters obtained from the two sequences showed strong correlations (the R-value ranged from 0.62 to 0.93). Both sequences showed that the craniocaudal peak velocity at C2-C3 was significantly higher than the caudocranial peak velocity (CINE-PC: 2.4 cm/s vs. 1.5 cm/s; RT-PC: 2.2 cm/s vs. 1.3 cm/s; *p* < 0.01). In addition, RT-PC significantly underestimated the peak velocity (*p* < 0.01). The velocity in the aqueduct was only 50% of that measured by CINE-PC.

**Table 1:** Comparison of CINE-PC and RT-PC measurements for CSF at C2-C3 level and at aqueduct

	C2-C3 (*n* = 32)			Aqueduct (*n* = 32)		
	CINE-PC	RT-PC	R-value	CINE-PC	RT-PC	R-value
Segment area (mm^2^)	148 ± 44 (131; 80–256)	165 ± 46 (150; 102–264)	0.91**	3.0 ± 0.8 (2.9; 1.7–5.1)	7.4 ± 1.8 (7.1; 3.2–10.2)	0.2
Q_net_ (ml/min) - Net flow rate of Qt	1.7 ± 8.6 (2.8; -20.1–17.5)	10 ± 4.8 (10; 0.6–22.9)	0.27	-0.47 ± 0.47 (-0.43; -1.8–0.3)	-0.03 ± 1.29 (-0.1; -5.0–3.1)	0
Amplitude (ml/min) - Amplitude flow rate	320 ± 58 (310; 158–465)	327 ± 74 (318; 191–577)	0.83**	19.3 ± 8.0 (19.3; 7.0–45.8)	22.1 ± 9.4 (21.5; 8.6–49.6)	0.93**
SV (10^− 3^ml) - Average Stroke volume	615 ± 151 (602; 270–923)	581 ± 152 (550; 307–901)	0.90**	43 ± 23 (36; 12–104)	46 ± 26 (40; 12–114)	0.89**
T_c_ (s) - Cardiac period of Q_t_	0.87 ± 0.17 (0.83; 0.56–1.39)	0.86 ± 0.16 (0.84; 0.54–1.37)	0.93**	0.87 ± 0.17 (0.83; 0.57–1.43)	0.86 ± 0.17 (0.83; 0.54–1.42)	0.89**
V_Max_ (cm/s) - Caudocranial peak velocity	1.5 ± 0.5 (1.5; 0.7–2.8)	1.3 ± 0.4 (1.2; 0.7–2.1)	0.85**	5.0 ± 1.7 (4.7; 2.2–8.7)	2.4 ± 1.1 (2.4; 0.7–4.9)	0.63**
V_Min_ (cm/s) - Craniocaudal peak velocity	2.4 ± 0.7 (2.3; 1.1–4.3)	2.2 ± 0.6 (2.2; 1.2–3.6)	0.89**	5.6 ± 1.5 (5.5; 3.3–8.9)	2.7 ± 1.2 (2.5; 0.2–4.8)	0.62**

Values are presented as mean ± standard deviation (SD), median, and range (min–max). The asterisks indicate the level of significance of the correlation for each parameter (* = *p* < 0.05; ** = *p* < 0.01). The R-values indicate the correlation coefficients

**Fig. 5:**
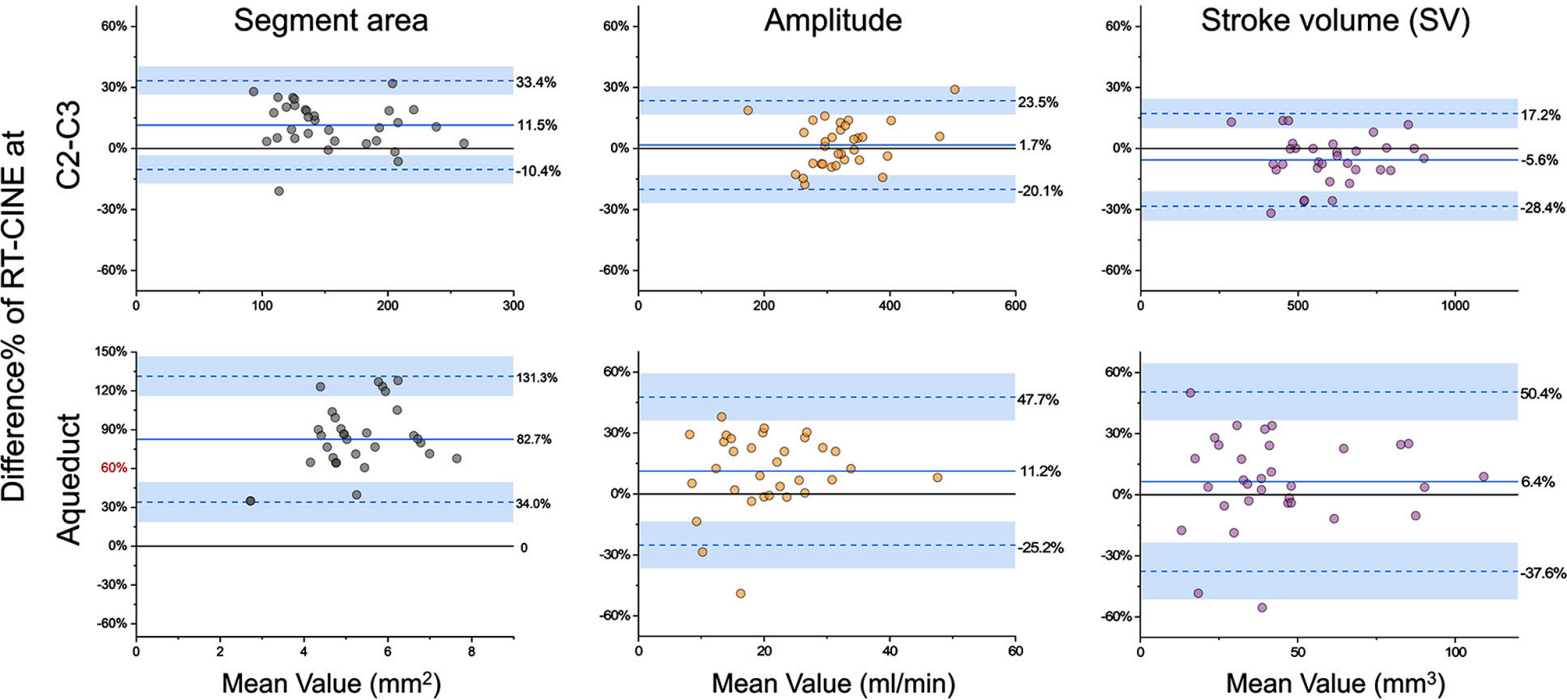
Bland-Altman plots illustrating the percentage differences between RT-PC and CINE-PC measurements for three CSF parameters (Segment area, Amplitude and stroke volume) at C2-C3 (top) and the aqueduct (bottom). The solid line represents the mean percentage difference, while the dashed lines indicate the limits of agreement (Mean ± 1.96 standard deviations)

The Bland-Altman plots in Fig. 5 show the percentage differences between RT-PC and CINE-PC for three CSF parameters: Segment area, Amplitude, and SV, at C2-C3 and aqueduct levels. These plots show that RT-PC overestimated the segment area at the aqueduct level by 82.7%. The differences in Amplitude were 1.7% at C2-C3 and 11.2% at the aqueduct. For the SV, the variations were within 10% for both planes, specifically − 5.6% at C2-C3 and 6.4% at the aqueduct. Notably, the aqueduct showed wider limits of agreement across all parameters.

### Aim 2: quantification of the breathing effects on CSF

From the initial 26 participants, one C2-C3 and five aqueduct datasets were excluded due to irregular breathing patterns or noise interference. Consequently, for Aim 2, we analyzed 25 datasets at the C2-C3 level and 20 at the aqueduct level, with 19 participants contributing data for both levels.

Table 2 shows the breathing effects on different parameters at the two planes. Notably, both *ΔQ*_*net*_*%* and *ΔAmplitude%* exhibit higher values at the aqueduct compared to the C2-C3 level, with values of 6.3% and 6.8% at C2-C3, respectively, and 11% each at the aqueduct.

The value *‘Δp%* without *Φp°’* reflects the percentage difference of the parameter *p* between the inspiratory and expiratory intervals without taking into account the phase shift (*Φp°* = 0). This approach tends to underestimate the *Δp%* on all parameters. In the aqueduct, neither *ΔAmplitude%* nor *ΔSV%*, without *Φp°*, showed a significant difference from 0, with p-values of 0.36 and 0.43, respectively.

**Table 2:** Quantitative results of the breathing effects on four parameters of CSF at extracranial (C2-C3) and intracranial (aqueduct) planes

	C2-C3 (*n* = 25)					
	Mean value	*Δp*	*Δp%*	*Φp°*	*Δp%* without *Φp°*	*p* value
Q_net_ (ml/min)	9.4 ± 5.6	20 ± 9.9	6.3 ± 3.1	33 ± 27	5.3 ± 2.8	**
Amplitude (ml/min)	338 ± 82	22 ± 9	6.8 ± 2.8	-11 ± 75	2.2 ± 3.7	**
SV (mm^3^)	608 ± 160	85 ± 56	14 ± 7.1	-36 ± 43	8.7 ± 6.4	**
T_c_ (ms)	832 ± 164	49 ± 28	6.0 ± 3.4	-64 ± 51	2.5 ± 3.4	**
	**Aqueduct (** ***n*** ** = 20)**					
	Mean value	*Δp*	*Δp%*	*Φp°*	*Δp%* without *Φp°*	*p* value
Q_net_ (ml/min)	-0.34 ± 0.93	2.8 ± 1.7	11 ± 6.8	58 ± 36	6.5 ± 5.7	**
Amplitude (ml/min)	26 ± 12	2.9 ± 1.8	11 ± 4.9	-80 ± 82	-1.6 ± 8.8	0.36
SV (mm^3^)	55 ± 34	9.0 ± 7.5	15 ± 5	-42 ± 60	1.9 ± 9.4	0.43
T_c_ (ms)	837 ± 154	51 ± 32	5.8 ± 3.4	-40 ± 47	3.4 ± 3.2	**

*Δp* indicates the difference between the inspiratory and expiratory phases of the corresponding parameter-*p*, *Δp%* is the percentage expression. *Δp%* without *Φp°* indicates the *Δp%* without taking into account the phase shift (*Φp°* = 0). The statistical significance (*p*-value) of *Δp%* without *Φp°* compared to zero is tested with t-test (* = *p* < 0.05; ** = *p* < 0.01)

**Fig. 6:**
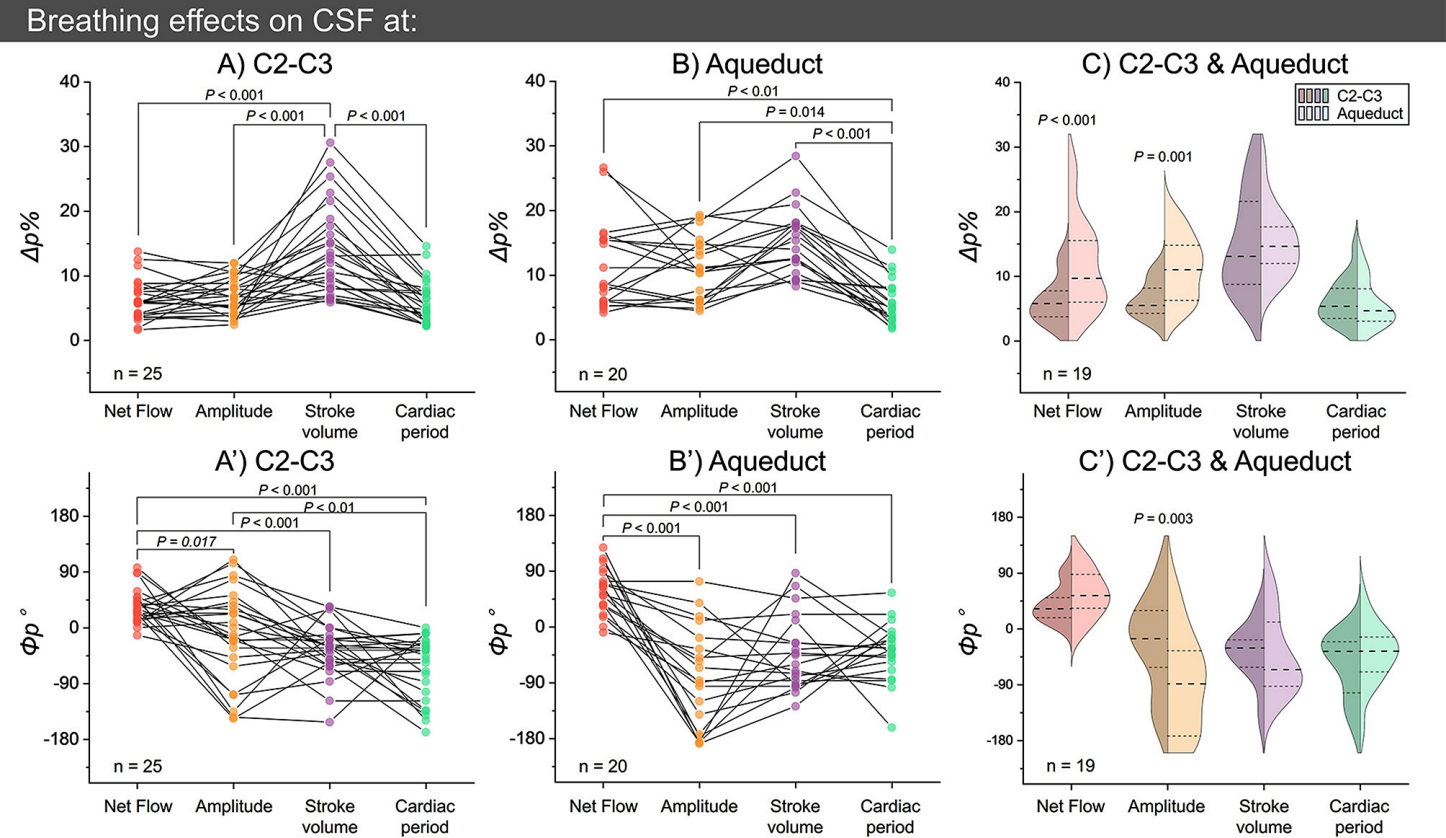
The intensity (*Δp%, top*) and corresponding phase shift (*Φp°, bottom*) of breathing effects on CSF parameters at C2-C3 (**A** and **A’**) and the aqueduct (**B** and **B’**). Paired t-tests or paired Wilcoxon tests were used to assess the significant differences between parameters (**A**, **A’**, **B** and **B’**) and levels (**C** and **C’**)

Figure 6 illustrates the distributions of breathing effects (*Δp%* and *Φp°*) for four CSF parameters in both planes. The *ΔSV%* is particularly higher compared to the other parameters (Fig. 6A and B). In addition, the *ΦQ*_*net*_*°* is significantly different from that of the other three parameters in both planes (Fig. 6A’ and Fig. 6B’). The *ΔQ*_*net*_*%* and *ΔAmplitude%* in the aqueduct were higher than in C2-C3 (Fig. 6C), with the *ΦAmplitude°* significantly differing between the two planes (Fig. 6C’).

**Fig. 7:**
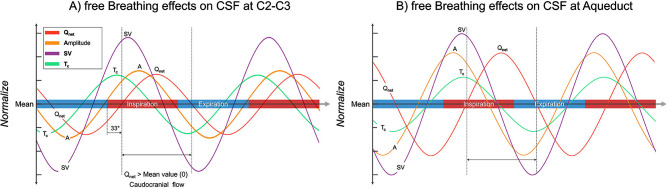
The curves of the CSF parameters under the influence of breathing are simulated by referring to the values of the breathing effects in Table 2. The inspiratory interval (0°–180°) is shown in red on the X-axis, indicating the process of increasing chest strap pressure, while the expiratory interval (180°–360°) is shown in blue. The middle line on the Y-axis represents the mean value of each parameter, taking into account that the mean of the net flow is 0 ml/min. The dashed interval represents the positive part of the net flow (Q_net_), indicating that the CSF is flowing towards the cranium. 33° in A) represents the *ΦQ*_*net*_*°*

Figure 7 displays a simulation of CSF parameter variations using the values from Table 2, providing a visual representation of the breathing effects on intensity and phase shifts in CSF parameters. In the figure, the SV appears to be influenced by Tc and Amplitude. In addition, all three parameters– SV, T_c_ and Amplitude– reach their maximum values as CSF begins to flow into the intracranial or third ventricle, coinciding with Q_net_ turning from negative to positive.

## Discussion

In this study, we assessed the accuracy of RT-PC in quantifying intracranial and extracranial CSF flow rates against the gold standard, CINE-PC. Our findings validate RT-PC’s precision in generating flow rate curves. Moreover, we detailed the free breathing effects on CSF parameters in both planes, emphasizing their intensity (*Δp%*) and corresponding phase shifts (*Φp°*).

### Aim 1: RT-PC vs. CINE-PC

In Aim 1, CSF parameters at the extracranial (C2-C3) and intracranial (aqueduct) levels were quantified using both CINE-PC and RT-PC. Our findings regarding Segment area and SV align with previous research [10, 11, 33]. At the C2-C3 level, both sequences indicated that caudal CSF flow velocities were significantly higher than cranial velocities (V_Min_ > V_Max_), as shown in Table 1. This higher caudal flow could be explained by the timing disparity between arterial inflow and venous outflow into the cranium, coupled with the pronounced pulsatility of the arterial flow. Systolic inflow occurs over one-third of the cardiac cycle and the diastolic period—which accounts for two-thirds—is primarily for venous outflow [47]. Therefore, the caudal CSF flow is more prone to aliasing.

Human heart rates are inherently variable. In our study, we observed a cardiac period variation exceeding 10% between RT-PC and CINE-PC measurements in 4 out of the 36 participants, leading to their exclusion (Figs. 3 and 4). The significant waveform discrepancies observed in the data (T22 in Fig. 3) may result from reconstruction errors linked to cardiac gating issues during acquisition, as illustrated in Fig. S2 & Fig. S3. To ensure clinical applicability of CINE-PC, repeat acquisition is necessary, given the impact of cardiac period variability and potential cardiac gating errors during acquisition.

CINE-PC, which requires cardiac synchronization, compiles multiple cardiac cycles to form a single averaged cycle, maintaining high pseudo-temporal [19]. Conversely, RT-PC, without synchronization, directly fills the k-space for each frame, with temporal resolution determined by the number of k-space segmentations and the TR. In our study, the RT-PC is a 4-shot RT-PC (number segmentation = Matrix acquisition / sensitivity encoding factor / EPI-factor = 70/2.5/7 = 4), and the temporal resolution was about 100 ms (temporal resolution = 2 × number segmentation × TR). This allows reconstruction of 10 points per cardiac cycle when the BPM is equal to 60. Thus, the accuracy of RT-PC quantification is affected by heart rate [30]. A previous in vitro study found that RT-PC can adapt to lower spatial resolution, although it has a lower signal-to-noise ratio than CINE-PC [48]. To enhance temporal resolution and signal-to-noise ratio, a lower spatial resolution is often employed in RT-PC. However, reduced spatial resolution may result in partial volume artifact [49], leading to an overestimation of the segmented area by RT-PC, especially in the aqueduct. Our study found that despite this overestimation in segment area, mean velocities decreased. Finally RT-PC’s flow rate curves closely matched that of CINE-PC (Figs. 3 and 4), with a difference of SV within 10% (Table 1: -5.6% in C2-C3 and 6.4% in aqueduct).

Increasing the VENC slightly is another way to increase temporal resolution. When the VENC falls below a certain threshold, the system extends the dual-gradient magnetic field duration, affecting TE and TR times [48]. In our study, most RT-PC acquisitions used relatively high VENC (10 cm/s for C2-C3 and 20 cm/s for aqueduct), which did not detrimentally affect the quantification’s accuracy. Moreover, this practice mitigated the aliasing problem to some extent and simplified post-processing.

Although numerous studies consider net flow (Q_net_) as a key metric, the validity of using phase contrast MRI to quantify CSF Q_net_ as a clinical benchmark remains controversial [11, 50–53]. This is because Q_net_ is very low [50] (approximately 0.3 ~ 0.4 ml/min) and is influenced by a variety of external factors such as: (1) Breathing effects: As illustrated in our aim 2, free breathing has a significant effect on Q_net_ (*ΔQ*_*net*_ at C2-C3 = 20 ml/min and *ΔQ*_*net*_ at aqueduct = 2.8 ml/min). Typically, CINE-PC data acquisition does not account for complete breathing cycles due to the lack of respiratory sensors. Although RT-PC can eliminate breathing effects through reconstruction using multiple complete respiratory cycles, as demonstrated by the reconstructed Q_t_ in this study, other influences remain. (2) Ultra-low frequency modulation: Current literature suggests that the CSF Q_net_ is additionally modulated by ultra-low frequency (< 0.1 Hz) components [54, 55], possibly related to cerebrovascular autoregulation. (3) Eddy currents: The impact of eddy currents on the measurement results must also be addressed. Although we could select static tissue close to the target for background field correction to mitigate this effect, the variability of the selection region and the distance between the static tissue and the target meant that the effect of eddy currents could not be completely eliminated. While the influence of various factors on Q_net_ may be minimal for larger arterial or venous flows, their impact on CSF Q_net_ is substantial. Our study identified a lack of correlation between CINE-PC and RT-PC in the measurement of Q_net_, as shown in Table 1. Consequently, the interpretation of CSF Q_net_, whether derived from RT-PC or CINE-PC, should be approached cautiously. It is more suitably considered to represent changing trends rather than an exact value for individual diagnosis.

According to Harvey Cushing’s theory - the third circulation, cerebrospinal fluid is produced by the choroid plexus and flows substantially towards the arachnoid granules [56–58]. However, it is unclear whether such secretion is constant and sustained. In this study, the Q_net_ was very small - less than 3% of the amplitude flow rate - and showed a large standard deviation. Given these factors, we believe that neither the CINE-PC nor the RT-PC protocol employed here can accurately measure such minute Q_net_ of CSF that approach background levels. Consequently, we are cautious about interpreting these findings as reliable indicators of physiological secretion. To achieve a more precise quantification of Q_net_, it is crucial to enhance spatial and temporal resolution and extend the acquisition duration to mitigate the impact of ultra-low frequency oscillations.

At the C2-C3 level, the mean Q_net_ of CINE-PC did not show a significant difference from zero, whereas the mean Q_net_ of RT-PC was significantly higher than zero. This may be due to the temporal resolution limitations of RT-PC leading to an underestimation of craniocaudal CSF flow rates with greater rates of velocity change. Although non-zero Q_net_ values were found in this study, they were significantly small compared to the amplitude.

In this study, the RT-PC image count was increased from 300 to 500 to reduce the impact of ultra-low frequency oscillations on Q_net_ and to allow for the exclusion of data from abnormal respiratory intervals, while still retaining sufficient data for further processing.

The comparison between CINE-PC and RT-PC validates RT-PC’s accuracy in quantifying CSF flows at the aqueduct and cervical spine levels. RT-PC provides stroke volume and amplitude measurements with precision comparable to CINE-PC while eliminating the need for cardiac synchronization and significantly reducing acquisition time.

### Aim 2: quantification of the breathing effects on CSF

While some studies have investigated the interaction between controlled breathing and CSF oscillations [28, 34, 59], comprehensive research appears to be needed on how free, unregulated breathing affects CSF dynamics. Free-breathing is the most physiologically representative state, and it is unlikely to influence CSF to the same extent as deep, controlled breathing. Therefore, assessing the effects of free breathing on CSF dynamics in healthy adults is highly valuable. This research could provide a baseline for future studies and clinical assessments and further clarify the influence of various respiratory patterns on cerebral circulation.

Most studies employing RT-PC to quantify the effects of breathing on CSF have utilized frequency domain analysis. While this method quantifies breathing effects by analyzing flow amplitude within the breathing frequency range, it does not consider the irregularities in breathing frequency and the associated phase shifts. Contrary to frequency domain analysis, our study employs a time-domain approach based on the cardiac cycle flow curve (Q_t_). This method involves identifying these Q_t_ in relation to the breathing cycle, thereby facilitating a comprehensive quantification of breathing effects (*Δp%*) on various CSF parameters and capturing the corresponding phase shifts (*Φp°*). Laganà’s recent work [33] emphasizes the importance of considering phase shifts when using time-domain methods. As shown in Table 2, neglecting the *Φp°* can lead to underestimating the *Δp%* or to statistically insignificant results.

The breathing effects on the cardiac period (*ΔT*_*c*_*%*), i.e., respiratory sinus arrhythmia, have been studied for years [60]. Consistent with historical data [61–64], our study confirms that the cardiac cycle lengthens during expiration and shortens during inspiration. Angelone et al. underscored the influence of breathing patterns and inspiratory volume on *ΔT*_*c*_*%* and *ΦT*_*c*_*°* (Fig. 3 in [60]). Therefore, it is crucial to consider *Φp°* when quantifying *Δp%* on the neurofluids flow dynamics. Using RT-PC in conjunction with the post-processing methods developed in this study may provide new directions for further investigation of the physiological mechanisms underlying respiratory sinus arrhythmia in the context of cerebral circulation.

During the transition from the early inspiratory to early expiratory phases, CSF Q_net_ reaches its maximum peak (Table 2: *ΔQ*_*net*_*%* = 6.3% with *ΦQ*_*net*_*°* = -33° at C2-C3 and *ΔQ*_*net*_*%* = 11% with *ΦQ*_*net*_*°* = -58° at the aqueduct). These findings indicate that CSF flows toward the cranial during inspiration (Fig. 7), consistent recent studies [34, 35] that found that CSF moves towards the cranial during deep inspiration. Laganà et al. also observed CSF flow rate variations during different breathing patterns, highlighting the presence of breathing spectral components in free breathing (Fig. 7 in [33]). Gutiérrez-Montes et al. observed CSF flow during guided normal breathing using respiratory gating-based CINE-PC and found that CSF flowed caudocranially during inspiration and craniocaudally during expiration [28].

The SV measures the oscillatory volume of CSF traversing a defined acquisition plane for each cardiac cycle, making it a crucial parameter for diagnostics, therapy, and research. In contrast, Q_net_ often approaches zero and is not influenced by Amplitude variations. This study appears to be the first to quantify free breathing effects on CSF Amplitude and SV. Our data suggest that the SV is more significantly affected by breathing (Table 2), with greater inter-individual variability in *ΔSV%* than in *ΔAmplitude%* and *ΔT*_*c*_*%* (Fig. 6), highlighting its potential as a marker for breathing-induced changes in CSF dynamics.

Figure 7 illustrates the temporal changes in CSF dynamics, showing that T_c_ begins to increase during early expiration and reaches its maximum in early inspiration. During this period, the Amplitude also increases, leading to a consequent rise in the SV. As CSF flow reverses direction (Q_net_ from negative to positive), the three parameters - T_c_, Amplitude, and SV - reach their maximum values. During the period when spinal CSF flows into the cranial and aqueduct CSF flows into the third ventricle (Q_net_ > 0), these three parameters gradually decrease.

During the cardiac cycle, CSF flow balances intracranial blood volume changes, influenced by cranio-spinal compliance. Prior research has focused on how free breathing independently impacts cerebral arterial and venous blood flows [65, 66]. Consequently, variations in CSF flow observed during breathing could be an indirect consequence of these changes in blood flow due to respiration. However, the precise mechanisms behind changes in intracranial cerebral blood volume during the cardiac cycle are still not fully understood. Factors such as arterial-venous flow delay, intracranial compliance, heart rate, gravity, and even breathing amplitude may indirectly influence cerebrospinal fluid flows. Consequently, CSF circulation is a highly complex phenomenon, and a better understanding of its physiological mechanisms may open the doors to new practices. It may be possible to actively influence CSF dynamics and apply this in clinical diagnostics by controlling the amplitude or period of respiration.

### Shortcomings and prospects

There are several limitations to this study. First, the narrow age distribution of the participants limited the analysis of breathing effects across age groups. Second, the analysis did not differentiate between thoracic and abdominal breathing, although previous studies have suggested significant differences in the breathing effect between the two patterns [34]. Future research could aim to quantify the effects of different breathing patterns on CSF, using the effects of free breathing as a reference.

Although RT-PC imaging is an invaluable tool, there are some technical limitations. Firstly, the temporal resolution limitation may affect the sampling points of Q_t_ due to the variability of the cardiac period, thereby affecting the accuracy of quantification. Secondly, the spatial resolution limitation prevents an accurate quantification of the breathing effects on the changes in vessel cross-sectional area. In addition, it is difficult to quantify cerebral blood and CSF flow rates simultaneously due to the large difference between their flow velocities. Despite these challenges, we are optimistic that emerging techniques such as compressed sensing and shared velocity encoding may overcome these barriers and open up new possibilities for RT-PC applications [67, 68].

## Conclusion

Real-time phase-contrast MRI (RT-PC) achieves a spatial resolution of 2 × 2 mm² and temporal resolution under 100 ms per image, enabling the continuous assessment of CSF flow dynamics over extended periods. The stroke volumes derived from RT-PC exhibited less than a 10% difference compared to those obtained via CINE-PC. No correlation was observed between the CSF net flow measured by RT-PC and CINE-PC, suggesting that CSF net flow is not recommended as a validated robust marker with the proposed RT-PC protocol.

Using a time-domain analysis method that accounts for phase shifts, we have quantified the effects of free breathing on various CSF flow parameters in both the aqueduct and cervical spinal level. All measured CSF flow parameters– net flow, amplitude, stroke volume, and cardiac period - increased during inspiration, with stroke volume showing the most significant impact by free breathing. Notably, the change of stroke volume between inspiration and expiration was more pronounced at the aqueduct than at the spinal level.

This study confirms the feasibility of using RT-PC for clinical application. The results of quantifying the effects of free breathing on CSF provide a valuable reference for further physiological studies to better understand the mechanisms of CSF circulation within the cranio-spinal compartments and its potential applications in investigating the physiopathology of idiopathic cerebral disorders.

### Electronic supplementary material

Below is the link to the electronic supplementary material.

**Supplementary Material 1: Figure 1.** Flowchart for selecting stationary tissues in the background field correction algorithm; **Figure 2.** CSF Qt data analysis for participant 22 at the C2-C3 level and prepontine cistern (PPC); **Figure 3.** Comparative analysis of cerebral blood flow (Qt-CBF) and CSF flow curves (Qt-CSF) for participant 22

## Data Availability

No datasets were generated or analysed during the current study.

## Abbreviations

*∆p*: The maximum difference of parameter p between the inspiratory and expiratory phases
*∆p%*: The maximum percentage difference of parameter p between the inspiratory and expiratory phases
*Φp°*: The corresponding phase shift degree to obtain the maximum *∆p* or *∆p%*
*Δp(Φp)*: The curve of *∆p* under different phase shift (*Φp*)
Amplitude: Amplitude flow rate
C2-C3: Second to third cervical vertebrae
CSF: Cerebrospinal fluid
CINE-PC: Conventional cine phase contrast MRI
FOV: Field of view
Q_net_: Mean value of Q_t_
Q_t_: Cardiac cycle flow rate curve
RT-PC: Real time phase contrast MRI
SV: Average Stroke volume
T_c_: Cardiac period of reconstructed or averaged Q_t_
VENC: Velocity encoding
V_Max_: Average velocity at the moment of maximum Q_t_ (cranial direction)
V_Min_: Average velocity at the moment of minimum Q_t_ (caudal direction)
