# Supplementary material for: Validating the accuracy of real-time phase-contrast MRI and quantifying the effects of free breathing on cerebrospinal fluid dynamics
Source: Fluids Barriers CNS. 2024 Mar 7;21:25. doi: 10.1186/s12987-024-00520-0 (PMC10921772; doi:10.1186/s12987-024-00520-0)
Supplement: Supplementary file 1 — Supplementary Material 1: Figure 1. Flowchart for selecting stationary tissues in the background field correction algorithm; Figure 2. CSF Qt data analysis for participant 22 at the C2-C3 level and prepontine cistern (PPC); Figure 3. Comparative analysis of cerebral blood flow (Qt-CBF) and CSF flow curves (Qt-CSF) for participant 22 [file 12987_2024_520_MOESM1_ESM.docx]

Additional data


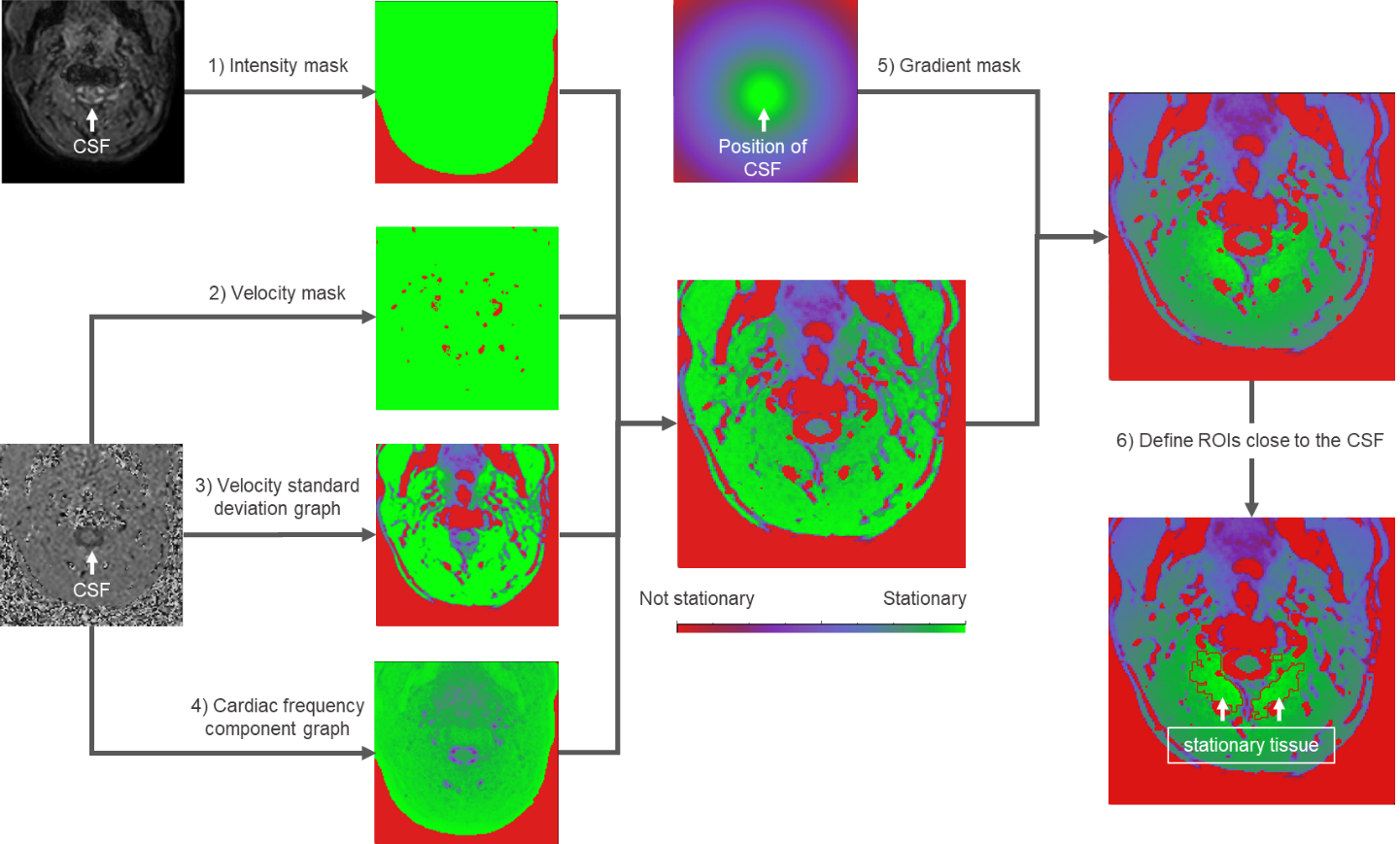


**Fig. S1 Flowchart for selecting stationary tissues in the background field correction algorithm.**

The process has the following steps:

1. Subsequent computation is reduced by removing air based on pixel intensity in amplitude images.
2. Analyze the average velocity of each pixel in the phase contrast image and discard blood vessels with absolute average velocities significantly above zero.
3. Exclude areas with high velocity standard deviations, which represent cavities or bone tissue.
4. Perform Fast Fourier Transformation on pixel velocity curves, removing regions with prominent cardiac frequency components, such as the region of CSF.
5. To create the final stationary tissue image, a gradient mask based on the target location is applied to the composite image generated in steps 1-4. The green area indicates stationary tissue near the CSF.
6. Automatically select stationary tissue adjacent to CSF.


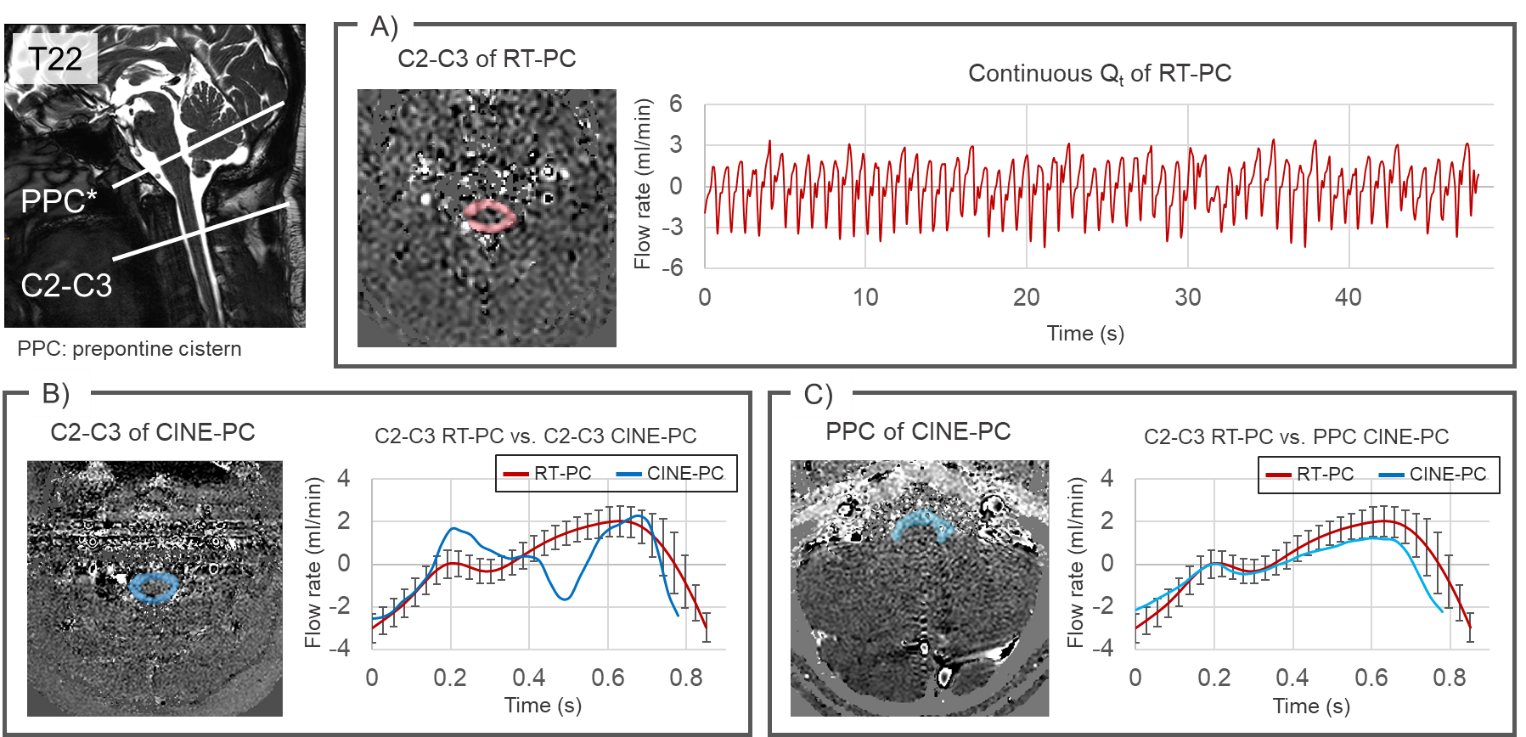


**Fig. S2 CSF Qt data analysis for participant 22 at the C2-C3 level and prepontine cistern (PPC).** A) Phase contrast image of RT-PC and extracted continuous Qt at the C2-C3 level. B) Comparative analysis of Qt from CINE-PC and reconstructed Qt from RT-PC at the C2-C3 level. C) Comparison between reconstructed Qt from RT-PC at the C2-C3 level and Qt from CINE-PC at the PPC level.

In this study, significant waveform differences were observed between RT-PC Q_t_-CSF and CINE-PC Q_t_-CSF at the C2-C3 level in participant 22. It is hypothesized that this difference is due to cardiac gating issues during CINE-PC acquisition, which may result in reconstruction errors. To confirm this hypothesis, CSF flow data from the prepontine cistern acquired by CINE-PC in the same study were analyzed. A significant similarity was observed when compared with RT-PC Q_t_-CSF (Fig. S2 C), which reinforces our hypothesis.

**
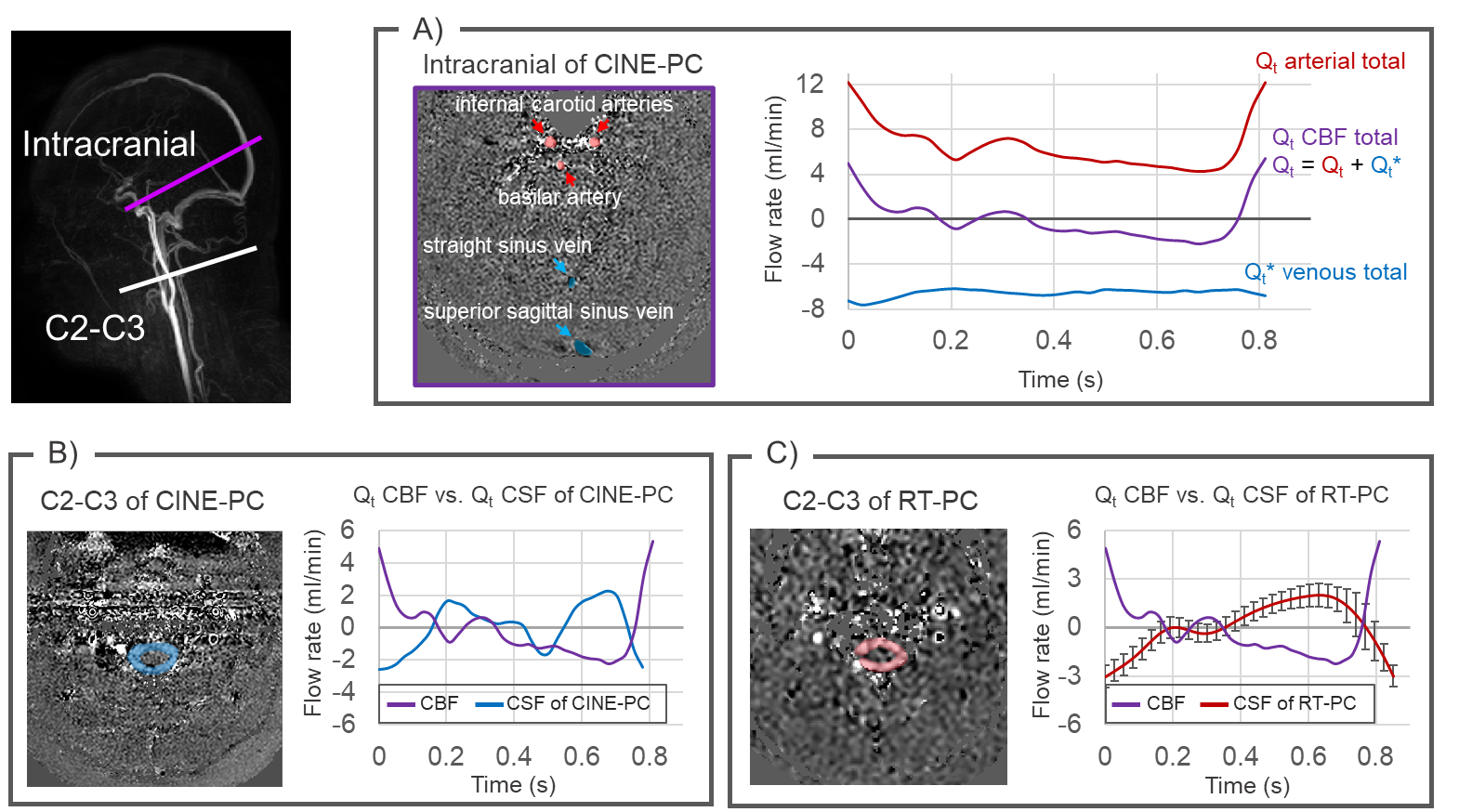
**

**Fig. S3 Comparative analysis of cerebral blood flow (Q_t_-CBF) and CSF flow curves (Q_t_-CSF) for participant 22.**  A) Total arterial flow (Q_t_-artery) of two internal carotid arteries and the basilar artery, and total venous flow (Q_t_-vein) of the straight sinus and superior sagittal sinus, the Q_t_-CBF calculated as Q_t_-artery plus λ*Q_t_-venous (λ represents the mean Q_t_-artery to Q_t_-vein ratio). B) Q_t_-CBF versus Qt-CSF of CINE-PC. C）Q_t_-CBF versus Q_t_-CSF of RT-PC.

To further validate our hypothesis, we calculated total cerebral blood flow (Q_t_-CBF) curves from arterial and venous flows in intracranial level, as shown in Fig. S3. As CBF drives CSF, there should be a strong symmetry between Q_t_-CBF and Q_t_-CSF. The results support our hypothesis that Q_t_-CSF from RT-PC shows greater symmetry with Q_t_-CBF than that from CINE-PC.

In clinical practice, if CINE-PC shows abnormal Q_t_ waveforms, reacquisition is advisable to eliminate potential sequence reconstruction errors due to cardiac gating problems.
